# New species of *Nipponoserica* and *Paraserica* from China (Coleoptera, Scarabaeidae, Sericini)

**DOI:** 10.3897/zookeys.721.13918

**Published:** 2017-12-12

**Authors:** Wan-Gang Liu, Silvia Fabrizi, Xingke Yang, Ming Bai, Dirk Ahrens

**Affiliations:** 1 Centre of Taxonomy and Evolutionary Research, Zoologisches Forschungsmuseum A. Koenig, Adenauerallee 160, 53113 Bonn, Germany; 2 Key Laboratory of Zoological Systematics and Evolution, Institute of Zoology, Chinese Academy of Sciences, Box 92, No. 1, Beichen West Road, Chaoyang District, Beijing, 100101, P.R. China; 3 Institute of Earth and Environment, Chinese Academy of Sciences, Yanxiang Road 97#, Yanta District, Xi’an 710061 P.R. China

**Keywords:** Beetles, chafers, China, new records, new species, *Nipponoserica*, *Paraserica*

## Abstract

The species of the genera *Nipponoserica* Nomura, 1973 and *Paraserica* Reitter, 1896 from China are revised. The following eight new species are described from China: *Paraserica camillerii* Ahrens, Fabrizi, & Liu, **sp. n.**, *P. mupuensis* Ahrens, Fabrizi, & Liu, **sp. n.**, *P. wangi* Ahrens, Fabrizi, & Liu, **sp. n.**, *Nipponoserica alloshanghaiensis* Ahrens, Fabrizi, & Liu, **sp. n.**, *N. anjiensis* Ahrens, Fabrizi, & Liu, **sp. n.**, *N. jiankouensis* Ahrens, Fabrizi, & Liu, **sp. n.**, *N. henanensis* Ahrens, Fabrizi, & Liu, **sp. n.**, and *N. sericanioides* Ahrens, Fabrizi, & Liu, **sp. n.** A key to the species of the genera examined here and maps of the species distribution are provided. Habitus and male genitalia are illustrated.

## Introduction

In the course of the revision of the Sericini of China a series of papers was published recently dealing mainly with the genera that possess a multi-lamellate antennal club (Ahrens et al. 2014a, b, c; Liu et al. 2014a, b, c, 2015; Liu et al. 2016). In continuation with this work, the results of the revision of the genera *Paraserica* Reitter, 1896 and *Nipponoserica* Nomura, 1973, both representing genera with a tri-lamellate antennal club, are presented here. So far, both groups are poorly known in China. While *Nipponoserica* is known only from four species, *Paraserica* is known from a single species from mainland China (Ahrens 2004b; Ahrens and Bezdek 2016). The examined material from different private and institutional collections contained a number of new species which are described herein. Furthermore, the material included numerous new and interesting locality records that are also given.

## Materials and methods

The terminology and methods used for measurements, specimen dissections, and genital preparation follow Ahrens (2004a). Data from specimens examined are cited in the text with original label contents given in quotation marks, multiple labels are separated by a “/”. Male genitalia were glued to a small pointed card attached to the specimen. Descriptions and illustrations of new taxa are based on the holotype or lectotype specimen, while the variation of other specimens is given separately. All descriptions and measurements were made under an Olympus SZX 12 microscope, and all genital and habitus illustrations were made with a digital camera (AxioCam HRc) attached to a stereo microscope (Zeiss Stereo Discovery V20) and Axio Version 4.8 software. The distribution maps were generated using Q-GIS 2.0.1 and Inscape software. Type specimens and other examined material are deposited in the following institutions or collections:

**CPPB** Coll. Petr Pacholátko, Brno, Czech Republic;

**HBUM** Museum of Hebei University, Baoding (Hebei Prov.), China;

**IZAS** Institute of Zoology, Chinese Academy of Sciences, Beijing, China;

**NUYS** Northwest A & F University, Yangling (Shaanxi Prov.), China;

**ZFMK** Zoologisches Forschungsmuseum A. Koenig, Bonn, Germany.

## Systematics

### Nipponoserica

Taxon classificationAnimaliaColeopteraScarabaeidae

Nomura, 1973

Nipponoserica Nomura, 1973b: 120 (type species: Serica similis Lewis, 1895 – by original designation; Nomura 1973b); Ahrens 2005: 276.
Pseudomaladera Nikolajev, 1980: 40 [homonym] (type species: Serica koltzei Reitter, 1897 – by original designation; Nikolajev 1980).

#### Key to the Chinese Nipponoserica species (♂♂).

**Table:** 

1	Dorsal surface shiny	**3**
–	Dorsal surface dull	**2**
2	Parameres distinctly asymmetrical	***N. elliptica* (Murayama, 1938)**
–	Parameres symmetrical	***N. koltzei* (Reitter, 1897)**
3	Penultimate abdominal sternite with a deep longitudinal furrow	**4**
–	Penultimate abdominal sternite without a deep longitudinal furrow	***N. sericanioides* Ahrens, Fabrizi, & Liu, sp. n.**
4	Distal portion of parameres symmetrical	**5**
–	Distal portion of parameres asymmetrical	***N. jiankouensis* Ahrens, Fabrizi, & Liu, sp. n.**
5	Dorsomedial sinuation of apical phallobase as deep as wide. Basal lobes of parameres bent forward and directed mesodistally	***N. sulciventris* Ahrens, 2004**
–	Dorsomedial sinuation of apical phallobase distinctly deeper than wide	**6**
6	Parameres with a small external tooth before apex	**7**
–	Parameres without small external tooth before apex	**8**
7	Sides of parameres convexly widened at middle	***N. shanghaiensis* Ahrens, 2004**
–	Sides of parameres concave at middle.	***N. anjiensis* Ahrens, Fabrizi, & Liu, sp. n.**
8	Each paramere with an internal short hook-like tooth	***N. alloshanghaiensis* Ahrens, Fabrizi, & Liu, sp. n.**
–	Paramere unarmed, without an internal tooth	**9**
9	Parameres longer, distinctly longer than phallobase at apex wide	***N. dahongshanica* Ahrens, 2005**
–	Parameres short, only litter longer than phallobase at apex wide	***N. henanensis* Ahrens, Fabrizi, & Liu, sp. n.**

### 

Checklist of the genus *Nipponoserica* and its occurrence (corrected, from Ahrens and Bezdek 2016; abbreviations: HUB – Hubei, GAN – Gansu, GUI – Guizhou, HEI – Heibei, HEN – Henan, JIX – Jiangxi, SCH – Sichuan, SHA – Shaanxi, SHG – Shanghai, ZHE – Zheijang, XIZ – Xizang, FE – Russian Far East, SC – South Korea, JA – Japan, TAI – Taiwan, NARi – Nearctic region, imported):

*Nipponoserica alloshanghaiensis* Ahrens, Fabrizi, & Liu, sp. n. A: JIX

*Nipponoserica anjiensis* Ahrens, Fabrizi, & Liu, sp. n. A: SHA, ZHE

*Nipponoserica babai* Kobayashi, 1991a: 47 A: TAI

*Nipponoserica chinensis* Moser, 1915b: 144 (as *Serica*) [doubtful assignment] A: SHN

*Nipponoserica daisensis* Sawada, 1937: 24 (as *Serica*) A: JA

syn. *lewisi* Chapin, 1938: 68 (as *Serica*)

*Nipponoserica dahongshanica* Ahrens, 2005: 276 A: HUB

*Nipponoserica elliptica* Murayama, 1938: 17 (as *Serica*) A: SC JIX

*Nipponoserica gomandana* Nomura, 1976: 187 A: JA

*Nipponoserica henanensis* Ahrens, Fabrizi, & Liu, sp. n. A: HEN

*Nipponoserica jiankouensis* Ahrens, Fabrizi, & Liu, sp. n. A: GUI

*Nipponoserica koltzei* Reitter, 1897: 214 (as *Serica*) A: FE GAN HEI HEN SHA XIZ SC

syn *opacicarina* Kim & Kim, 2003a: 76

*Nipponoserica kunitachiana* Nomura, 1976b: 190 A: JA

*Nipponoserica laferi* Nikolajev, 1980: 41 (*Pseudomaladera*) A: FE

*Nipponoserica peregrina* Chapin, 1938: 68 (*Serica*) A: JA NARi

*Nipponoserica pubiventris* Nomura, 1976: 189 A: JA

*Nipponoserica sericanioides* Ahrens, Fabrizi, & Liu, sp. n. A: ZHE

*Nipponoserica shanghaiensis* Ahrens, 2004: 8 A: SHG

*Nipponoserica similis* Lewis, 1895c: 391 (as *Serica*) A: JA

syn *setiventris* Nomura, 1976: 188

*Nipponoserica sulciventris* Ahrens, 2004: 9 A: GAN HUB SCH SHA

*Nipponoserica takeuchii* Hirasawa, 1991: 171 [doubtful assignment] A: TAI

### Nipponoserica elliptica

Taxon classificationAnimaliaColeopteraScarabaeidae

(Murayama, 1938)

Figures 2I–L , 4

Serica elliptica Murayama, 1938: 17; Kim and Lee 1991: 55, 60 (fig. 7a,b).
Nipponoserica elliptica : Nomura 1973: 139; Murayama 1954: 20.

#### Material examined.

**South Korea**: 1 ♂ “27.06.2010 Beomeosa, Busan (Südkorea) leg. T. Kölkebeck” (ZFMK). **China**: 1 ♂ “Yiyang, Jiangxi, 16.V.1975, leg. Zhang Youwei” (IZAS), 1 ♂ “Yiyang, Jiangxi, 13.V.1975, leg. Zhang Youwei” (IZAS).

#### Redescription.

Length: 9.6 mm, length of elytra: 7.6 mm, width: 5.1 mm. Body oblong, including legs dark brown, antenna yellowish brown, dorsal surface dull and glabrous.

*Labroclypeus* subtrapezoidal and wide, widest at base; lateral margins convex and strongly convergent, with weakly rounded anterior angles; lateral border and ocular canthus producing a distinct blunt angle; margins weakly reflexed; anterior margin distinctly sinuate medially; surface flat and weakly shiny, finely and densely punctate, with a few short, erect setae anteriorly.

*Frontoclypeal* suture indistinctly incised and weakly curved medially; smooth area in front of eye 1.5 times as wide as long; ocular canthus short and triangular, finely and sparsely punctate with a short single terminal seta. Frons dull, with fine and moderately dense punctures, with a few long setae beside eyes. Eyes moderately large, ratio of diameter/interocular width: 0.7. Antenna with nine antennomeres; club with three antennomeres, 1.3 times as long as remaining antennomeres combined, weakly reflexed. Mentum elevated and flattened anteriorly. Labrum produced and deeply sinuate medially.

*Pronotum* transverse, widest at base, lateral margins moderately convex and convergent anteriorly; anterior angles weakly produced and blunt; posterior angles blunt, rounded at tip; anterior margin strongly and convexly produced medially with a distinct and broad marginal line; basal margin without marginal line; hypomeron distinctly margined at base; surface with moderately dense and fine punctures, glabrous; anterior and lateral borders sparsely setaceous. Scutellum narrow and long, well pointed at apex, with fine and moderately dense punctures.

*Elytra* oblong, widest in posterior third, striae distinctly impressed, finely and densely punctate; intervals convex, with fine and sparse punctures concentrated along striae, glabrous; epipleural border robust, ending at strongly curved external apical angle; epipleura densely setaceous; apical border chitinous with a fine rim of microtrichomes (visible at 100× magnification).

*Ventral surface* dull, metasternum partly shiny, with moderately dense, large punctures, sparsely setose, only on metasternal disc with a few longer setae. Metacoxa glabrous, laterally with a few fine setae. Abdominal sternites with fine, dense punctation, each with indistinct transversal row of coarse punctures bearing a short seta; penultimate sternite with a shallow and short median furrow. Mesosternum between mesocoxae half as wide as mesofemur, with irregularly scattered, strong setae. Ratio of length of metepisternum/ metacoxa: 1/1.4. Pygidium dull, weakly convex, finely and moderately densely punctate, without smooth midline, glabrous except a few longer setae on apical half.

*Legs* slender, dull; femora with two longitudinal rows of setae, finely and moderately densely punctate. Metafemur dull, anterior margin acute, without a submarginal serrated line; posterior margin straight with a few strong setae medially, ventrally weakly widened in apical half and serrate; dorsally serrated with short setae. Metatibia slender and long, widest shortly before apex, ratio width/length: 1/4.6; dorsal margin sharply carinate, with one group of spines (basal group of spines reduced) at four-fifths of metatibial length, basally with a few single spines in punctures; external face beside dorsal margin longitudinally roof-like carinate, sparsely finely punctate, with some longitudinal, superficial wrinkles; ventral margin finely serrate, with four fine nearly equidistant setae; medial face impunctate but superficially wrinkled; apex bluntly truncate interiorly near tarsal articulation. Tarsomeres glabrous and impunctate dorsally, with sparse, short setae ventrally; metatarsomeres with a strongly serrated ridge ventrally and a fine longitudinal carina laterally; first metatarsomere slightly shorter than following two combined, slightly longer than dorsal tibial spur. Protibia moderately long, bidentate, protarsal claws symmetrical. Aedeagus: apical part of parameres asymmetrical, basal lobes symmetrical.

#### Remarks.

We were unable to locate and examine the type material of this species; possibly the types are lost. Specimens were identified according to Kim and Lee (1991).

#### Distribution.

The species was known only from South Korea (Ahrens and Bezdek 2016). Now it is recorded for the first time from China, Jiangxi province.

### Nipponoserica koltzei

Taxon classificationAnimaliaColeopteraScarabaeidae

(Reitter, 1897)

Figure 4

Serica koltzei Reitter, 1897: 214.
Nipponoserica koltzei : Nomura 1973: 139; Nikolaev 2002: 98; Ahrens 2004b: 7.
Pseudoserica koltzei : Nikolaev 1980: 40.
Nipponoserica opacicarina Kim & Kim, 2003: 76; syn by Ahrens 2007: 9.

#### Additional material examined.

2 ex. “China, W Henan, 9.VII.2006 Funiu Shan, 33°42'N, 112°15'E Shirenshan 1400–1900m, Jaroslav Turna leg. “(ZFMK), 17 ex. “China, W Henan, 6–7.VII.2006 Funiu Shan, 33°31'N, 111°56'E Baotianman, 1500–1750m, Jaroslav Turna leg.” (ZFMK), 1 ex. “China-Shaanxi, SW Tsinling Mts., Taiping vill., 33°33'N, 106°43'E, June 2000, 1500–2000m, Siniaev & Plutenko leg.” (CPPB), 1 ex. “China-Shaanxi, Tsinling Mts., Houzhenzi vill., 33°53'N, 107°49'E, June-Juli 2000, 1500m, Siniaev & Plutenko leg.” (CPPB), 2 ex. “China, 1000–1300m, Shaanxi, Qinling mts., Xunyangba (6km E) 23.v.-13.vi.1998 J.H. Marshal leg.” (CPPB), 1 ex. “China, W Henan, 7.–8.VII.2007, Funju Shan, N33°42', E112°15', Shirenshan, 1500m, leg. Jaroslav Turna” (ZFMK), 1 ♂ “Bayi, Xizang, No.255” (IZAS), 1 ♂ “Ha’erbin, 16.VI.1960” (IZAS), 3 ♂♂ “Getiaopa, Neixiang, Henan, 15.VII.1998, 600m, leg. Zhang Youwei” (IZAS).

#### Distribution.

The species was known from South Korea, and Far East of Russia, as well as Gansu and Hubei provinces of China (Ahrens and Bezdek 2016). It is now recorded for the first time from Henan, Shaanxi, and Xizang provinces of China.

### Nipponoserica sulciventris

Taxon classificationAnimaliaColeopteraScarabaeidae

Ahrens, 2004

Figure 4

Nipponoserica sulciventris Ahrens, 2004b: 9.

#### Additional material examined.

2 ex. “China, W Hubei prov., Dashennogjia Nat. Res., Muyu, E slope, 2000 m, 12–15 Jun 1997, Bolm lgt.” (CPPB), 2 ex. “China, N Sichuan, 5.–6.VI. Micang Shan, 1300–1400m, Daba, 32°40'N 106°55'E Jaroslav Turna leg., 2007” (ZFMK), 6 ex. “China, W Hubei, 21.–24.VI. Dashennongjia mts. 31.5N 110.3E, 2500–3000m Jaroslav Turna leg. 2001” (CPPB), 1 ♂ “Gansu, 7.IX.1980s” (HBUM).

#### Distribution.

The species was known so far from Hubei, Shaanxi and Sichuan provinces of China (Ahrens and Bezdek 2016). It is now recorded for the first time also from Gansu province (China).

##### New taxa

### Nipponoserica anjiensis

Taxon classificationAnimaliaColeopteraScarabaeidae

Ahrens, Fabrizi, & Liu sp. n.

http://zoobank.org/020469E7-21C6-4A89-9A97-9DFE89722C91

Figures 1A–D , 4

#### Type material examined.

Holotype: ♂ “Mts. Longwangshan, Anji, Zhejiang, 12.V.1996, 450m, leg. Wu Hong/ LW-236” (IZAS). Paratypes: 1 ♂ “Wugong, Shaanxi, 2.VI.1974/ LW-085” (NUYS), 1 ♂ “Bayi, 29.VI.1982” (IZAS).

#### Diagnosis.

*Nipponoserica anjiensis* sp. n. is very similar to *N. shanghaiensis* Ahrens in external appearance but differs in having distinctly shorter parameres. Sides of parameres concave at middle.

**Figure 1.**
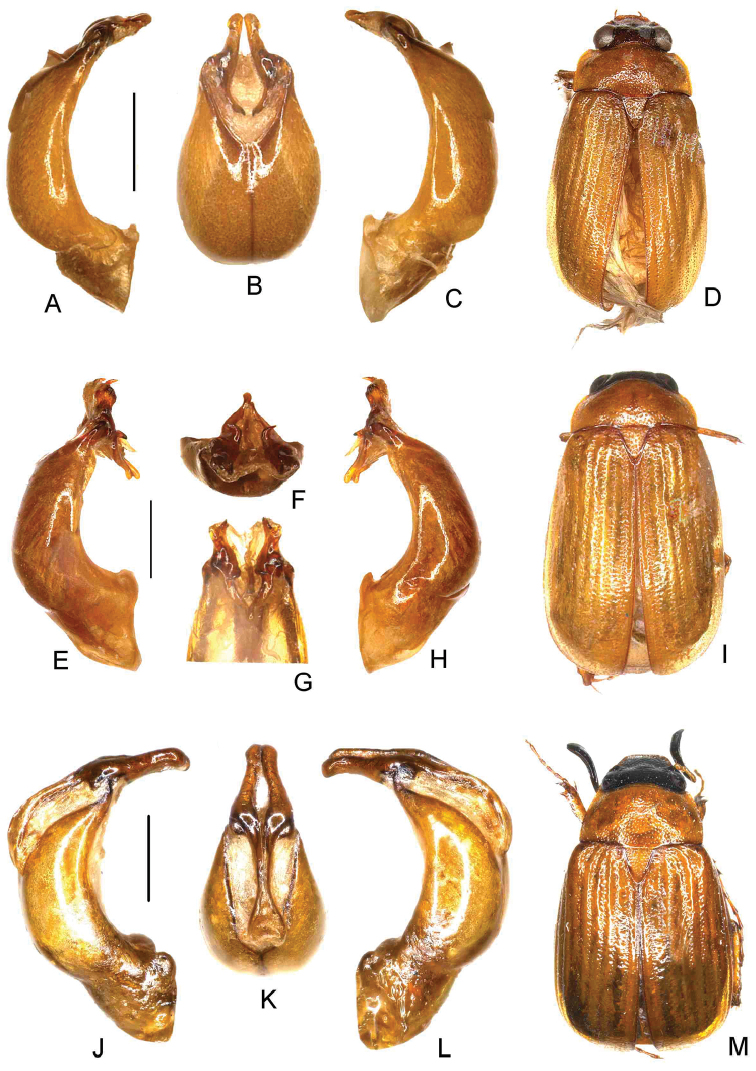
**A–D** *Nipponoserica anjiensis* Ahrens, Fabrizi, & Liu, sp. n. (holotype) **E–I** *N. alloshanghaiensis* Ahrens, Fabrizi, & Liu, sp. n. (holotype) **J–M** *N. henanensis* Ahrens, Fabrizi, & Liu, sp. n. (holotype) **A, E, J** aedeagus, left side lateral view **C, H, L** aedeagus, right side lateral view **B, G, K** parameres, dorsal view **F** parameres, basidorsal view **D, I, M** habitus. Scale bar 0.5 mm. Habitus not to scale.

#### Description.

Length: 8.5 mm, length of elytra: 6.4 mm, width: 3.8 mm. Body oblong, yellow, frons darker brown, antenna yellowish brown, dorsal surface entirely shiny and glabrous.

*Labroclypeus* subtrapezoidal and moderately wide, widest at base; lateral margins weakly convex and moderately convergent with moderately rounded anterior angles, lateral border and ocular canthus producing a distinct blunt angle; margins weakly reflexed; anterior margin distinctly sinuate medially; surface weakly convex medially and shiny, finely and very densely punctate, distance between punctures less than their diameter, anteriorly with a few long, erect setae. Frontoclypeal suture feebly incised and medially weakly angled; smooth area in front of eye short, approximately 2.5 times as wide as long; ocular canthus moderately long and slender, finely and densely punctate with a short single terminal seta. Frons with fine and sparsely but regularly scattered punctures, with a few short setae beside the eyes. Eyes very large, ratio of diameter/interocular width: 0.94. Antenna with nine antennomeres; club with three antennomeres, almost 3 times as long as remaining antennomeres combined, strongly reflexed. Mentum elevated and flattened anteriorly. Labrum slightly produced and deeply sinuate medially.

*Pronotum* wide and transverse, widest shortly before base, lateral margins weakly convex and weakly narrowed anteriorly; anterior angles moderately produced and blunt, posterior angles right angled but strongly rounded at tip; anterior margin strongly and convexly produced medially with a distinct and broad marginal line; basal margin without marginal line; hypomeron distinctly margined at base; surface with moderately dense and fine punctures, with microscopic setae in punctures, otherwise glabrous; anterior and lateral borders sparsely setaceous. Scutellum narrow and long, well pointed at apex, with fine and moderately dense punctures.

*Elytra* oblong, widest in posterior third, striae distinctly impressed, finely and densely punctate; intervals weakly convex, with fine and dense punctures concentrated along striae, glabrous except for a few fine setae on penultimate lateral interval; epipleural border robust, ending at strongly curved external apical angle; epipleura densely setaceous; apical border chitinous with a very fine rim of short microtrichomes (visible at 100× magnification).

*Ventral surface* partly dull or shiny, with dense, large punctures, sparsely setose, only metasternal disc with a few longer setae. Metacoxa glabrous, laterally with a few fine setae. Abdominal sternites with fine, dense punctation, each with indistinct transversal row of coarse punctures bearing a short seta; penultimate sternite with a shallow and short median furrow. Mesosternum between mesocoxae almost as wide as mesofemur, with irregularly scattered, strong setae. Ratio of length of metepisternum/ metacoxa: 1/1.3. Pygidium dull and weakly convex, finely and moderately densely punctate, without smooth midline, with sparse short setae and a few longer setae adjacent to apical margin.

*Legs* slender, shiny; femora with two longitudinal rows of setae, finely and moderately densely punctate. Metafemur shiny, anterior margin acute, without a submarginal serrated line; posterior margin straight with a few strong setae medially, ventrally weakly widened in apical half and serrate; dorsally serrated with short setae. Metatibia slender and long, widest shortly before apex, ratio width/length: 1/3.9; dorsal margin sharply carinate, with one group of spines (basal group of spines reduced) at four-fifths of metatibial length, basally with a few single spines in punctures; external face beside dorsal margin longitudinally roof-like carinate, impunctate but with some longitudinal, very superficial wrinkles; ventral margin finely serrate, with three fine setae, of which the two apical are more distant; medial face impunctate but superficially wrinkled; apex bluntly truncate interiorly near tarsal articulation. Tarsomeres glabrous and impunctate dorsally, with sparse, short setae ventrally; metatarsomeres dorsally with weak longitudinal impressions, ventrally with a strongly serrated ridge and a fine longitudinal carina laterally; first metatarsomere little longer than second, one third of its length longer than the upper tibial spur. Protibia moderately long, bidentate, protarsal claws symmetrical. Aedeagus: Parameres symmetrical, with asymmetrical basal lobes.

#### Etymology.

The new species is named after the type locality Anji.

#### Variation.

Length: 8.0–8.5 mm, length of elytra: 6.0–6.4 mm, width: 3.6–3.8 mm.

### Nipponoserica alloshanghaiensis

Taxon classificationAnimaliaColeopteraScarabaeidae

Ahrens, Fabrizi, & Liu sp. n.

http://zoobank.org/142DDDCC-6FB5-456F-8163-DA1C60923E7B

Figures 1E–I , 4

#### Type material examined.

Holotype: ♂ “Mts. Lushan, 10.V.1977, leg. Zhang Youwei” (IZAS). Paratypes: 3 ♀♀ “Mts. Lushan, 10.V.1977, leg. Zhang Youwei” (IZAS, ZFMK).

#### Diagnosis.

*Nipponoserica alloshanghaiensis* sp. n. has distinctly longer parameres; each parameres has a sharp median tooth in the middle (at the level of its insertion to phallobase).

#### Description.

Length: 7.8 mm, length of elytra: 6.4 mm, width: 4.6 mm. *Body* oblong, yellow, frons darker brown, antenna yellowish brown, dorsal surface moderately shiny and glabrous.

Labroclypeus subtrapezoidal and moderately wide, widest at base; lateral margins weakly convex and moderately convergent with moderately rounded anterior angles, lateral border and ocular canthus producing a distinct blunt angle; margins weakly reflexed; anterior margin distinctly sinuate medially; surface weakly convex medially and shiny, finely and very densely punctate, distance between punctures less than their diameter, anteriorly with a few long, erect setae. Frontoclypeal suture feebly incised and medially weakly angled; smooth area in front of eye short, approximately 2.5 times as wide as long; ocular canthus moderately long and slender, finely and densely punctate with a short single terminal seta. Frons with fine and sparsely but regularly scattered punctures, with a few short setae beside eyes. Eyes large, ratio of diameter/interocular width: 0.83. Antenna with nine antennomeres; club with three antennomeres, three times as long as remaining antennomeres combined, strongly reflexed. Mentum elevated and flattened anteriorly. Labrum slightly produced and deeply sinuate medially.

*Pronotum* wide and transverse, widest shortly before base, lateral margins weakly convex and weakly narrowed anteriorly; anterior angles moderately produced and blunt, posterior angles right angled but strongly rounded at tip; anterior margin strongly and convexly produced medially with a distinct and broad marginal line; basal margin without marginal line; hypomeron distinctly margined at base; surface with moderately dense and fine punctures, with microscopic setae in punctures, otherwise glabrous; anterior and lateral borders sparsely setaceous. Scutellum narrow and long, well pointed at apex, with fine and moderately dense punctures.

*Elytra* oblong, widest in posterior third, striae distinctly impressed, finely and densely punctate; intervals weakly convex, with fine and dense punctures concentrated along striae, glabrous except for a few fine setae on penultimate lateral interval; epipleural border robust, ending at strongly curved external apical angle; epipleura densely setaceous; apical border chitinous with a very fine rim of short microtrichomes (visible at 100x magnification).

*Ventral surface* dull or shiny, with moderately dense, large punctures, sparsely setose, only metasternal disc with a few longer setae. Metacoxa glabrous, laterally with a few fine setae. Abdominal sternites with fine, dense punctation, each with indistinct transversal row of coarse punctures bearing a short seta; penultimate sternite with a shallow and short median furrow, apical margin of sternite tooth-like elevated beside furrow. Mesosternum between mesocoxae half as wide as mesofemur, with irregularly scattered, strong setae. Ratio of length of metepisternum/ metacoxa: 1/1.2. Pygidium dull, moderately convex, finely and moderately densely punctate, without smooth midline, with sparse short setae and a few longer setae adjacent to apical margin.

*Legs* slender, shiny; femora with two longitudinal rows of setae, finely and moderately densely punctate. Metafemur shiny, anterior margin acute, without a submarginal serrated line; posterior margin straight with a few strong setae medially, ventrally weakly widened in apical half and serrate; dorsally serrated with short setae. Metatibia slender and long, widest shortly before apex, ratio width/length: 1/4.5; dorsal margin sharply carinate, with one group of spines (basal group of spines reduced) at four-fifths of metatibial length, basally with a few single spines in punctures; external face beside dorsal margin longitudinally roof-like carinate, impunctate but with some longitudinal, very superficial wrinkles; ventral margin finely serrate, with three fine setae, of which the two apical are more distant; medial face impunctate but superficially wrinkled; apex bluntly truncate interiorly near tarsal articulation. Tarsomeres glabrous and impunctate dorsally, with sparse, short setae ventrally; metatarsomeres dorsally with weak longitudinal impressions, ventrally with a strongly serrated ridge and a fine longitudinal carina laterally; first metatarsomere little longer than second, one third of its length longer than the upper tibial spur. Protibia moderately long, bidentate, protarsal claws symmetrical.

#### Etymology.

The name of the new species is combined from the Greek prefix *allo*- (other than) and the species name *shanghaiensis*, with reference to the very similar *Nipponoserica shanghaiensis* Ahrens.

#### Variation.

Length: 7.8–8.8 mm, length of elytra: 6.4–7.1 mm, width: 4.6–4.9 mm. Female: Antennal club short, slightly shorter than remaining antennomeres combined; eyes small, ratio of diameter/interocular width: 0.58.

### Nipponoserica henanensis

Taxon classificationAnimaliaColeopteraScarabaeidae

Ahrens, Fabrizi, & Liu sp. n.

http://zoobank.org/28D1A818-5DFE-48CD-A4E3-0F262AD42B83

Figures 1J–M , 4

#### Type material examined.

Holotype: ♂ “China, W Henan, 15.V.–2.VI. Funiu Shan, Baotianman, pitfall traps, 33.5N 111.9E Jaroslav Turna leg., 2005” (ZFMK). Paratypes: 1 ♂, 3 ♀♀ “China, W Henan, 16.–18.V. Funiu Shan, 33°31'N 111°56'E Baotianman, pitfall traps, 1500–1750 m Jaroslav Turna leg., 2008” (ZFMK).

#### Diagnosis.

*Nipponoserica henanensis* sp. n. has distinctly shorter and more robust parameres than *N. dahonshanica* Ahrens, which are distinctly shorter than their basal lobe.

#### Description.

Length: 7.7 mm, length of elytra: 5.8 mm, width: 4.6 mm. *Body* oblong, yellow, frons blackish, labroclypeus and ventral surface dark brown, antenna yellowish brown, dorsal surface shiny and glabrous.

*Labroclypeus* subtrapezoidal and moderately wide, widest at base; lateral margins straight and moderately convergent with weakly rounded anterior angles, lateral border and ocular canthus producing a distinct blunt angle; margins weakly reflexed; anterior margin moderately but broadly sinuate medially; surface flat and shiny, finely and very densely punctate, with a few long, erect setae anteriorly. Frontoclypeal suture indistinctly incised and weakly curved medially; smooth area in front of eye twice as wide as long; ocular canthus moderately short and triangular, finely and densely punctate with a short single terminal seta. Frons with fine and dense punctures, with a few short setae beside eyes. Eyes small, ratio of diameter/interocular width: 0.52. Antenna with nine antennomeres; club with three antennomeres, 2.3 times as long as remaining antennomeres combined, strongly reflexed. Mentum elevated and flattened anteriorly. Labrum produced and deeply sinuate medially.

*Pronotum* narrow, widest at base, lateral margins weakly convex and weakly narrowed anteriorly; anterior angles weakly produced but sharp; posterior angles blunt; anterior margin strongly and convexly produced medially with a distinct and broad marginal line; basal margin without marginal line; hypomeron distinctly margined at base; surface with dense and fine punctures, glabrous; anterior and lateral borders sparsely setaceous. Scutellum narrow and long, well pointed at apex, with fine and moderately dense punctures.

*Elytra* oblong, widest in posterior third, striae distinctly impressed, finely and densely punctate; intervals weakly convex, with fine and sparse punctures concentrated along striae, glabrous except for a few short setae on odd intervals; epipleural border robust, ending at strongly curved external apical angle; epipleura densely setaceous; apical border chitinous with a fine rim of short microtrichomes (visible at 100× magnification).

*Ventral surface* dull, metasternum partly shiny, with moderately dense, large punctures, sparsely setose, only on metasternal disc with a few longer setae. Metacoxa glabrous, laterally with a few fine setae. Abdominal sternites with fine, dense punctation, each with indistinct transversal row of coarse punctures bearing a short seta; penultimate sternite with a shallow and short median furrow, apical margin of sternite tooth-like elevated beside furrow. Mesosternum between mesocoxae half as wide as mesofemur, with irregularly scattered, strong setae. Ratio of length of metepisternum/ metacoxa: 1/1.35. Pygidium shiny, apical half dull, moderately convex, finely and moderately densely punctate, without smooth midline, with sparse short setae and a few longer setae adjacent to apical margin.

*Legs* slender, shiny; femora with two longitudinal rows of setae, finely and moderately densely punctate. Metafemur shiny, anterior margin acute, without a submarginal serrated line; posterior margin straight with a few strong setae medially, ventrally weakly widened in apical half and serrate; dorsally serrated with short setae. Metatibia slender and long, widest shortly before apex, ratio width/length: 1/4.2; dorsal margin sharply carinate, with one group of spines (basal group of spines reduced) at five-sixths of metatibial length, basally with a few single spines in punctures; external face beside dorsal margin longitudinally roof-like carinate, sparsely finely punctate, with some longitudinal, superficial wrinkles; ventral margin finely serrate, with three fine equidistant setae; medial face impunctate but superficially wrinkled; apex bluntly truncate interiorly near tarsal articulation. Tarsomeres glabrous and impunctate dorsally, with sparse, short setae ventrally; metatarsomeres with a strongly serrated ridge ventrally and a fine longitudinal carina laterally; first metatarsomere distinctly longer than second, one third of its length longer than the upper tibial spur. Protibia moderately long, bidentate, protarsal claws symmetrical.

#### Etymology.

The new species is named after its occurrence in the Henan province.

#### Variation.

Length: 7.7–8.2 mm, length of elytra: 5.8–6.0 mm, width: 4.6–4.7 mm. Female: Antennal club short, slightly shorter than remaining antennomeres combined; eyes small, ratio of diameter/interocular width: 0.43.

### Nipponoserica jiankouensis

Taxon classificationAnimaliaColeopteraScarabaeidae

Ahrens, Fabrizi, & Liu sp.n.

http://zoobank.org/249FAEC3-53AA-4771-ACE2-DE2C8514BB15

Figures 2A–D , 4

#### Type material examined.

Holotype: ♂ “CH-Guizhou NE 27.V.–3.VI. 20km NW of Jiangkou, 1995 Fanjing Shan-Kuaichang E. Jendek & O. Sausa leg./ Coll. P. Pacholatko Invt. No./ CS11” (CPPB).

#### Diagnosis.

*Nipponoserica jiankouensis* sp. n. has symmetrical basal lobes but an asymmetrical distal portion of parameres, with the right paramere being bent externally.

**Figure 2.**
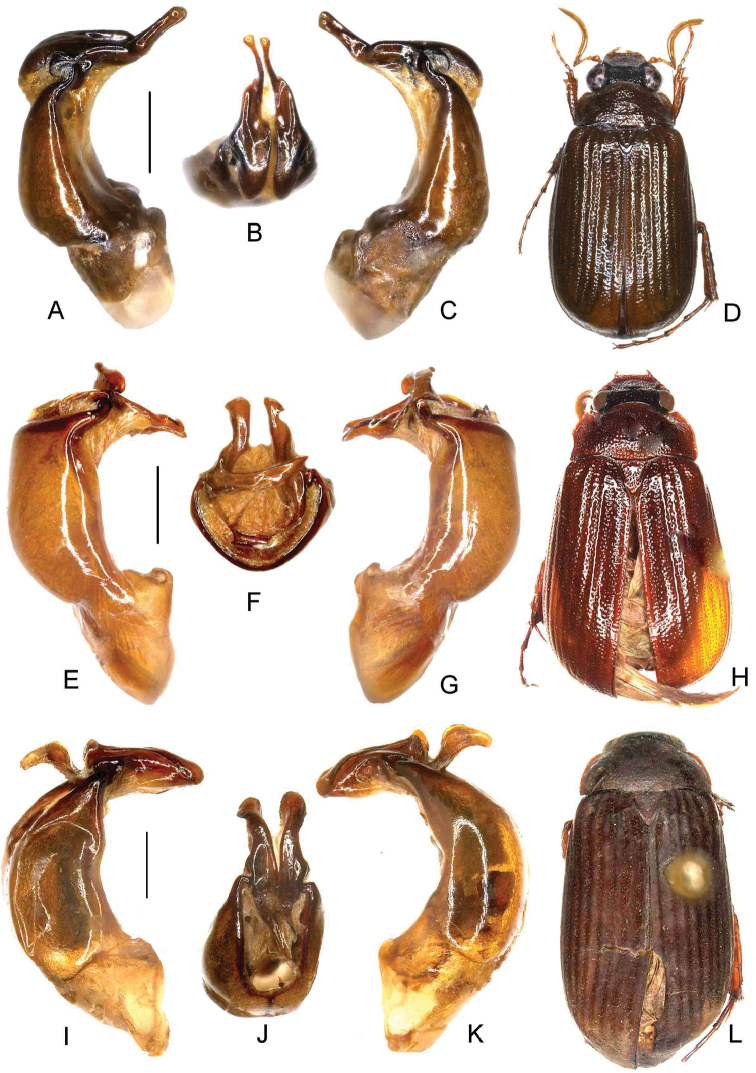
**A–D** *Nipponoserica jiankouensis* Ahrens, Fabrizi, & Liu, sp. n. (holotype) **E–H** *N. sericanioides* Ahrens, Fabrizi, & Liu, sp. n. (holotype) **I–L** *N. elliptica* (Murayama, 1938) (China: Yiyang, Jiangxi) **A, E, I** aedeagus, left side lateral view **C, G, K** aedeagus, right side lateral view **B, F, J** parameres, dorsal view **D, H, L** habitus. Scale bar 0.5 mm. Habitus not to scale.

#### Description.

Length: 7.8 mm, length of elytra: 5.6 mm, width: 4.1 mm. Body oblong, including legs dark yellow brown, frons and ventral surface dark brown, antenna yellowish brown, dorsal surface shiny and glabrous.

*Labroclypeus* subtrapezoidal and moderately wide, widest at base; lateral margins moderately convex and convergent, with moderately rounded anterior angles; lateral border and ocular canthus producing a distinct blunt angle; margins weakly reflexed; anterior margin distinctly sinuate medially; surface flat and shiny, finely and densely punctate, with a few short, erect setae anteriorly. Frontoclypeal suture indistinctly incised and weakly curved medially; smooth area in front of eye twice as wide as long; ocular canthus moderately short and triangular, finely and densely punctate with a short single terminal seta. Frons with fine and dense punctures, with a few short setae beside eyes. Eyes moderately large, ratio of diameter/interocular width: 0.74. Antenna with nine antennomeres; club with three antennomeres, three times as long as remaining antennomeres combined, strongly reflexed. Mentum elevated and flattened anteriorly. Labrum produced and deeply sinuate medially.

*Pronotum* transverse, widest at base, lateral margins straight and subparallel, in anterior quarter weakly convex and narrowed anteriorly; anterior angles weakly produced and blunt; posterior angles blunt, rounded at tip; anterior margin strongly and convexly produced medially with a distinct and broad marginal line; basal margin without marginal line; hypomeron distinctly margined at base; surface with dense and fine punctures, glabrous; anterior and lateral borders sparsely setaceous. Scutellum narrow and long, well pointed at apex, with fine and moderately dense punctures.

*Elytra* oblong, widest in posterior third, striae distinctly impressed, finely and densely punctate; intervals weakly convex, with fine and sparse punctures concentrated along striae, glabrous except for a few short setae on odd intervals; epipleural border robust, ending at strongly curved external apical angle; epipleura densely setaceous; apical border chitinous with a very fine rim of short microtrichomes (visible at 100x magnification).

*Ventral surface* dull, metasternum partly shiny, with moderately dense, large punctures, sparsely setose, only on metasternal disc with a few longer setae. Metacoxa glabrous, laterally with a few fine setae. Abdominal sternites with fine, dense punctation, each with indistinct transversal row of coarse punctures bearing a short seta; penultimate sternite with a shallow and short median furrow, apical margin of sternite tooth-like elevated beside furrow. Mesosternum between mesocoxae half as wide as mesofemur, with irregularly scattered, strong setae. Ratio of length of metepisternum/ metacoxa: 1/1.23. Pygidium completely dull, moderately convex, finely and moderately densely punctate, without smooth midline, with sparse short setae and a few longer setae on apical half.

*Legs* slender, shiny; femora with two longitudinal rows of setae, finely and moderately densely punctate. Metafemur shiny, anterior margin acute, without a submarginal serrated line; posterior margin straight with a few strong setae medially, ventrally weakly widened in apical half and serrate; dorsally serrated with short setae. Metatibia slender and long, widest shortly before apex, ratio width/length: 1/4.2; dorsal margin sharply carinate, with one group of spines (basal group of spines reduced) at five-sixths of metatibial length, basally with a few single spines in punctures; external face beside dorsal margin longitudinally roof-like carinate, sparsely finely punctate, with some longitudinal, superficial wrinkles; ventral margin finely serrate, with three fine setae of which the apical one is more distant; medial face impunctate but superficially wrinkled; apex bluntly truncate interiorly near tarsal articulation. Tarsomeres glabrous and impunctate dorsally, with sparse, short setae ventrally; metatarsomeres with a strongly serrated ridge ventrally and a fine longitudinal carina laterally; first metatarsomere distinctly longer than second, nearly twice as long as dorsal tibial spur. Protibia moderately long, bidentate, protarsal claws symmetrical. Aedeagus: Apical part of parameres asymmetrical, basal lobes symmetrical. Female unknown.

#### Etymology.

The new species is named after its occurrence in vicinity of Jiankou.

### Nipponoserica sericanioides

Taxon classificationAnimaliaColeopteraScarabaeidae

Ahrens, Fabrizi, & Liu sp. n.

http://zoobank.org/8F661A6E-6995-40FF-85BE-6D2975C72A5E

Figures 2E–I , 4

#### Type material examined.

Holotype: ♂ “Zheijang, Fengyangshan, Datianping, 2007-V-30/ LW-1242” (ZFMK). Paratypes: 1 ♂ “Zheijang, Fengyangshan, Datianping, 2007-V-30/ LW-1242bis” (IZAS).

#### Diagnosis.

*Nipponoserica sericanioides* sp. n. bears strong asymmetrical parameres (both the basal and distal portions), which somewhat resembles the general morphology of species of *Sericania*.

#### Description.

Length: 9.5 mm, length of elytra: 6.2 mm, width: 5.3 mm. *Body* oblong, including legs reddish brown, frons dark brown, antenna yellowish brown, dorsal surface shiny and glabrous.

*Labroclypeus* subtrapezoidal and moderately wide, widest at base; lateral margins moderately convex and convergent, with moderately rounded anterior angles; lateral border and ocular canthus producing a distinct blunt angle; margins weakly reflexed; anterior margin distinctly sinuate medially; surface slightly concave and shiny, finely and densely punctate, with a few short, erect setae anteriorly. Frontoclypeal suture indistinctly incised and weakly curved medially; smooth area in front of eye 1.5 times as wide as long; ocular canthus moderately short and triangular, finely and densely punctate with a short single terminal seta. Frons with fine and moderately dense punctures, with a few long setae beside eyes. Eyes small, ratio of diameter/interocular width: 0.6. Antenna with nine antennomeres; club with three antennomeres, 3 times as long as remaining antennomeres combined, strongly reflexed. Mentum elevated and flattened anteriorly. Labrum produced and deeply sinuate medially.

*Pronotum* transverse, widest at base, lateral margins straight and slightly convergent, in anterior third convex and narrowed anteriorly; anterior angles weakly produced and blunt; posterior angles blunt, rounded at tip; anterior margin strongly and convexly produced medially with a distinct and broad marginal line; basal margin without marginal line; hypomeron distinctly margined at base; surface with dense and fine punctures, glabrous; anterior and lateral borders sparsely setaceous. Scutellum narrow and long, well pointed at apex, with fine and moderately dense punctures.

Elytra oblong, widest in posterior third, striae distinctly impressed, finely and densely punctate; intervals weakly convex, with fine and sparse punctures concentrated along striae, glabrous except for a few short setae on odd intervals; epipleural border robust, ending at strongly curved external apical angle; epipleura densely setaceous; apical border chitinous without rim of microtrichomes (visible at 100× magnification).

*Ventral surface* dull, metasternum partly shiny, with moderately dense, large punctures, sparsely setose, only on metasternal disc with a few longer setae. Metacoxa glabrous, laterally with a few fine setae. Abdominal sternites shiny, with fine, dense punctation, each with indistinct transversal row of coarse punctures bearing a short seta; penultimate sternite with a shallow and short median furrow, apical margin of sternite tooth-like elevated beside furrow. Mesosternum between mesocoxae half as wide as mesofemur, with irregularly scattered, strong setae. Ratio of length of metepisternum/ metacoxa: 1/1.23. Pygidium shiny, at apex dull, moderately convex, finely and moderately densely punctate, without smooth midline, with sparse short setae and a few longer setae on apical half.

*Legs* slender, shiny; femora with two longitudinal rows of setae, finely and moderately densely punctate. Metafemur shiny, anterior margin acute, without a submarginal serrated line; posterior margin straight with a few strong setae medially, ventrally weakly widened in apical half and serrate; dorsally serrated with short setae. Metatibia slender and long, widest shortly before apex, ratio width/length: 1/3.9; dorsal margin sharply carinate, with one group of spines (basal group of spines reduced) at four-fifths of metatibial length, basally with a few single spines in punctures; external face beside dorsal margin longitudinally roof-like carinate, sparsely finely punctate, with some longitudinal, superficial wrinkles; ventral margin finely serrate, with three fine setae of which the apical one is more distant; medial face impunctate but superficially wrinkled; apex bluntly truncate interiorly near tarsal articulation. Tarsomeres glabrous and impunctate dorsally, with sparse, short setae ventrally; metatarsomeres with a strongly serrated ridge ventrally and a fine longitudinal carina laterally; first metatarsomere distinctly longer than second, distinctly longer than dorsal tibial spur. Protibia moderately long, bidentate, protarsal claws symmetrical. The aedeagus has the apical part of the parameres asymmetrical, but the basal lobes are symmetrical (Fig. 2E–G). Female unknown.

#### Etymology.

The name of the new species, according to its similarity to the species of the genus *Sericania*, is based on the genus name *Sericania* and the Greek suffix -*oides* (similar).

#### Variation.

Length: 9.5–10.0 mm, length of elytra: 6.2–7.1 mm, width: 5.2–5.3 mm.

### Paraserica

Taxon classificationAnimaliaColeopteraScarabaeidae

Reitter, 1896

Paraserica Reitter, 1896: 183 (type species: Serica grisea Motschulsky, 1866 by monotypy)

#### Key to the species (♂♂).

**Table:** 

1	Antennal club less than three times as long as remaining antennomeres combined	**2**
–	Antennal club four times as long as remaining antennomeres combined	***P. camillerii* Ahrens, Fabrizi, & Liu, sp. n.**
2	Species from Taiwan	***P. taiwana* Nomura & Kobayashi, 1979**
–	Species from Japan and mainland China	**3**
3	Antennal club more than twice as long as remaining antennomeres combined. Dorsomedial sinuation of apical phallobase as deep as wide	**4**
–	Antennal club twice as long as remaining antennomeres combined. Dorsomedial sinuation of apical phallobase distinctly deeper than wide	***P. mupuensis* Ahrens, Fabrizi, & Liu, sp. n.**
4	Apex of phallobase without lateral processes	***P. grisea* (Motschulsky, 1866)**
–	Apex of phallobase on each side bearing a lateral process	***P. wangi* Ahrens, Fabrizi, & Liu, sp. n.**

### Paraserica grisea

Taxon classificationAnimaliaColeopteraScarabaeidae

(Motschulsky, 1866)

Figure 5

Serica grisea Motschulsky, 1866: 171; Brenske 1897: 424, 1902: 48; Niijima and Kinoshita 1923: 15; Sawada 1937: 12.
Paraserica grisea : Reitter 1896: 183; Nakane and Baba 1960: 5; Nomura 1963: 125, 1966: 74, 1973: 124; Nakane 1972: 426; Ahrens 2004b: 7, 2007: 31.

#### Additional material examined.

1 ex. “Tsumbame-spa Myoko-Mts. Niigata-pref. 25.VII.1992 S. Tsuyuki leg.” (ZFMK), 1 ex. “Japan Kioto” (ZFMK), 1 ex. “China: Shaanxi 21.-23.VI.1998 Quing Ling Shan mts., road Baoji-Taibai pass 35km S of Baoji O. Safranek & M. Trycna leg.” (ZFMK), 1 ex. “China, W Hubei, 20.V. 5km S Lúcongpo 30.8N 110.25E Jaroslav Turna leg., 2004” (ZFMK), 4 ex. “China: Shaanxi prov., 21.–23. June 1998 Quing Ling Shan road Baoji-Tabai vill. pass. 40km S Baoji Zd. Jindra lgt.” (ZFMK), 2 ex. “China, W Hubei, 3.V.–15.VII. Muyuping NW env. 31°27'N, 110°22'E, 1600m Jaroslav Turna leg., 2006” (ZFMK), 1 ♂, 1 ♀ “Mts. Tsukuba, 12.VII.1932” (IZAS).

#### Distribution.

The species is distributed in Japan and newly recorded for the Chinese provinces of Hubei and Shaanxi.

### Paraserica taiwana

Taxon classificationAnimaliaColeopteraScarabaeidae

Nomura & Kobayashi, 1979

Paraserica taiwana Nomura & Kobayashi, 1979: 11.

#### Distribution.

The species occurs in the central mountainous part (Nantou County) of Taiwan.

#### Remarks.

Species is known (from Taiwan) only from female, thus the true identity of this species is uncertain yet. We had no chance yet to examine the holotype. Given its occurrence (Nantou County) in the central mountainous part of Taiwan which is characterised by a high endemism, it is highly improbable that this species does also occur in mainland China.

### Paraserica camillerii

Taxon classificationAnimaliaColeopteraScarabaeidae

Ahrens, Fabrizi, & Liu sp. n.

http://zoobank.org/A1CC1553-CBC3-48B1-9359-3CF6F7729971

Figures 3A–D , 5

#### Type material examined.

Holotype: “China: W Guizhou prov.; Leigongshan; Xijing; 29.v.-2.vi.1997; BOLM leg.; 1200–1900m/ 712 Sericini Asia sp.” (CPPB). Paratypes: 3 ♀♀ “China, W Guizhou prov. Leigongshan, Xijing 29 May - 2 Jun 1997 1200–1900m, BOLM lgt.” (CPPB, ZFMK), 1 ♂ “Mts. Leigongshan, Leishan, Guizhou, 2.VII.1988, 1700 m, leg. Zhang Xiaochun” (IZAS).

#### Diagnosis.

*Paraserica camillerii* sp. n. has distinctly asymmetric parameres with large basal lobes.

**Figure 3.**
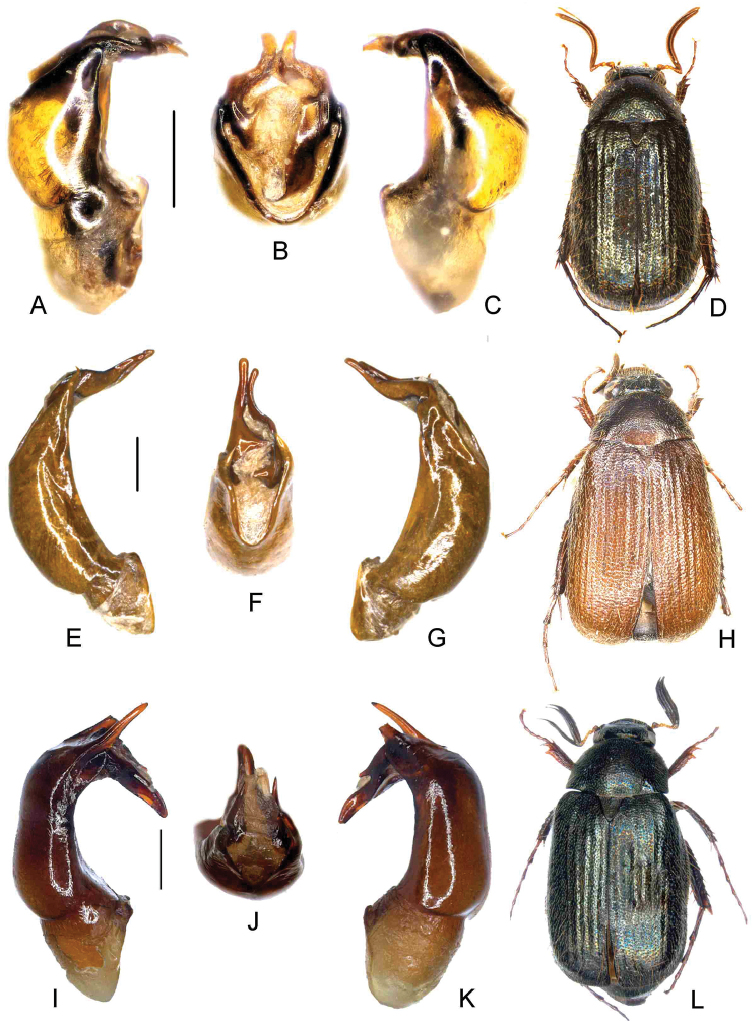
**A–D** *Paraserica camillerii* Ahrens, Fabrizi, & Liu, sp. n. (holotype) **E–H** *P. mupuensis* Ahrens, Fabrizi, & Liu, sp. n. (holotype) **I–L** *N. wangi* Ahrens, Fabrizi, & Liu, sp. n. (holotype) **A, E, I** aedeagus, left side lateral view **C, G, K** aedeagus, right side lateral view **B, F, J** parameres, dorsal view **D, H, L** habitus. Scale bar 0.5 mm. Habitus not to scale.

#### Description.

Length: 7.0 mm, length of elytra: 5.2 mm, width: 3.8 mm. *Body* including legs oblong, dark brown to grey-blackish, antenna yellowish brown, dorsal and ventral surface shiny and densely setose with double pilosity.

*Labroclypeus* subtrapezoidal and moderately wide, widest at base; lateral margins straight and moderately convergent, with moderately rounded anterior angles; lateral border and ocular canthus producing a distinct blunt angle; margins weakly reflexed; anterior margin nearly straight; surface flat and shiny, densely punctate, coarse punctures mixed with small ones; with long, dense, erect setae. Frontoclypeal suture indistinctly incised and weakly angled medially; smooth area in front of eye as wide as long; ocular canthus moderately short and narrow, finely and densely punctate, with a few setae. Frons with fine and dense punctures, with numerous short, adjacent setae and a few erect longer ones beside eyes and behind frontoclypeal suture. Eyes small, ratio of diameter/interocular width: 0.58. Antenna with nine antennomeres; club with three antennomeres, 4 times as long as remaining antennomeres combined, strongly reflexed. Mentum weakly elevated and flattened anteriorly. Labrum weakly produced and moderately sinuate medially.

*Pronotum* narrow, widest at base, lateral margins straight and subparallel in basal half, at middle moderately convex, again straight and strongly convergent in anterior half; anterior angles weakly produced but sharp; posterior angles right-angled; anterior margin straight, with a distinct and broad marginal line; basal margin without marginal line; hypomeron distinctly margined at base; surface with dense and fine punctures, with dense, double pilosity: numerous short setae bent backwards mixed with more sparse, long and erect setae; anterior and lateral borders densely setaceous. Scutellum narrow and long, with fine and dense punctures, with dense short setae.

*Elytra* oblong, widest shortly behind middle, striae distinctly impressed, finely and densely punctate; intervals weakly convex, with fine and sparse punctures, densely setose as the pronotum, long erect setae only on odd intervals; epipleural border robust, ending at strongly curved external apical angle; epipleura densely setaceous; apical border chitinous with a fine rim of short microtrichomes (visible at 100× magnification).

*Ventral surface* shiny, with moderately dense, fine punctures, with dense short adjacent setae. Metacoxa completely finely setose as rest of ventral surface, laterally with a few longer setae. Abdominal sternites with fine, dense punctation and short fine pilosity, each sternite with a distinct transversal row of coarse punctures bearing a long seta; penultimate sternite simple. Mesosternum between mesocoxae as wide as mesofemur, with irregularly scattered, strong setae. Ratio of length of metepisternum/ metacoxa: 1/1.5. Pygidium basally shiny, apical half dull, moderately convex, finely and moderately densely punctate, without smooth midline, with dense, moderately long setae and a few longer setae adjacent to apical margin.

*Legs* slender, shiny; femora with two longitudinal rows of setae, finely and moderately densely punctate, shortly sparsely setose. Metafemur shiny, anterior margin acute, without a submarginal serrated line; posterior margin straight with a few strong setae medially, ventrally weakly widened in apical half and smooth; dorsally serrated. Metatibia slender and long, widest shortly before apex, ratio width/length: 1/3.8; dorsal margin sharply carinate, with two groups of spines, basal on shortly behind middle, apical one at four-fifths of metatibial length, basally with a few single spines; external face longitudinally convex, densely coarsely punctate, with dense short setae; ventral margin finely serrate, with four robust equidistant setae; medial face impunctate but coarsely punctate along the inner dorsal and ventral margin, punctures each bearing a fine seta; apex moderately truncate interiorly near tarsal articulation. Tarsomeres densely punctate dorsally, with sparse, short setae ventrally; metatarsomeres with a strongly serrated ridge ventrally; first metatarsomere distinctly longer than second, one third of its length longer than the upper tibial spur. Protibia moderately long, bidentate, protarsal claws symmetrical.

#### Variation.

Length: 7.0–8.1 mm, length of elytra: 5.2–5.6 mm, width: 3.8–4.1 mm. Female: Antennal club with three antennomeres, as long as remaining antennomeres combined; eyes as large as in male.

#### Etymology.

This new species is dedicated to the Sicilian writer, Andrea Camillieri, whose books accompanied D.A.’s work on Chinese Sericini over all the years.

### Paraserica mupuensis

Taxon classificationAnimaliaColeopteraScarabaeidae

Ahrens, Fabrizi, & Liu sp. n.

http://zoobank.org/E584DF19-12BF-4930-A0DF-8DE70D4BB5C6

Figures 3E–H , 5

#### Type material examined.

Holotype: “China: Hunan; Mupu Mt. 1600m, Pingjiang VIII-2003, leg. Li et al.” (ZFMK). Paratypes: 5 ♂♂, 15 ♀♀ “China: Hunan; Mupu Mt. 1600m, Pingjiang VIII-2003, leg. Li et al.” (ZFMK), 1 ♂ “Jiugongshan Tongshan, S-Hubei, 1.V.2004, Leg Wen” (ZFMK), 4 ♂♂, 13 ♀♀ “China: Hubei, Dahongshan 1700m Shuizhou VI-2003 leg. Ying et al.” (ZFMK, CPPB, IZAS), 1 ♂ “Mts. Tienmushan, 12.VI.1936, leg. O. Piel, Musee Heude” (IZAS).

#### Diagnosis.

*Paraserica mupuensis* sp. n. has distinctly asymmetric parameres and the phallobase on each side of its apex with a narrow process.

#### Description.

Length: 8.8 mm, length of elytra: 6.2 mm, width: 4.6 mm. *Body* oblong, head and pronotum including legs dark brown, elytra reddish brown, antenna yellowish brown, dorsal and ventral surface shiny and densely setose with partly double pilosity.

*Labroclypeus* subtrapezoidal and wide, widest at base; lateral margins straight and moderately convergent, with moderately rounded anterior angles; lateral border and ocular canthus producing a distinct blunt angle; margins weakly reflexed; anterior margin distinctly sinuate medially; surface flat and moderately shiny, densely punctate, very coarse punctures mixed with small ones; with long, dense, erect setae. Frontoclypeal suture distinctly incised and weakly curved medially, slightly elevated; smooth area in front of eye 1.5 times as wide as long; ocular canthus moderately short and narrow, finely and densely punctate, with a 1–2 setae. Frons with fine and dense punctures, with numerous short, adjacent setae and a few erect longer ones beside eyes and behind frontoclypeal suture. Eyes small, ratio of diameter/interocular width: 0.64. Antenna with nine antennomeres; club with three antennomeres, 1.8 times as long as remaining antennomeres combined, strongly reflexed. Mentum elevated and flattened anteriorly. Labrum weakly produced and moderately sinuate medially.

*Pronotum* narrow, widest at base, lateral margins straight and weakly convergent in basal half, weakly convex and moderately convergent in anterior half; anterior angles weakly produced but nearly blunt; posterior angles right-angled; anterior margin weakly convex, with a distinct and broad marginal line; basal margin without marginal line; hypomeron distinctly margined at base; surface with dense and fine punctures, with dense, double pilosity: numerous short setae bent backwards mixed with very sparse, long and erect setae, at disc pilosity partly abraded; anterior and lateral borders densely setaceous. Scutellum narrow and long, with fine and dense punctures, with dense short setae.

*Elytra* oblong, widest shortly behind middle, striae distinctly impressed, finely and densely punctate; intervals weakly convex, with fine and sparse punctures, densely setose with short adjacent setae as pronotum, long erect setae absent on elytra; epipleural border robust, ending at strongly curved external apical angle; epipleura densely setaceous; apical border chitinous with a broad rim of short microtrichomes (visible at 100× magnification).

*Ventral surface* shiny, with moderately dense, fine punctures, with dense short adjacent setae. Metacoxa completely finely setose as rest of ventral surface, laterally with a few longer setae. Abdominal sternites with fine, dense punctation and short fine pilosity, each sternite with a distinct transversal row of coarse punctures bearing a long seta; penultimate sternite simple. Mesosternum between mesocoxae as wide as mesofemur, with irregularly scattered, strong setae. Ratio of length of metepisternum/ metacoxa: 1/1.5. Pygidium shiny, in apical half strongly convex, finely and densely punctate, without smooth midline, with dense, moderately long setae and numerous longer setae adjacent to apical margin.

*Legs* moderately slender, shiny; femora with two longitudinal rows of setae, finely and moderately densely punctate, shortly sparsely setose. Metafemur shiny, anterior margin acute, without a submarginal serrated line; posterior margin with a few strong setae medially, weakly widened in apical half and smooth ventrally; finely serrated dorsally. Metatibia slender and long, widest shortly before apex, ratio width/length: 1/3.6; dorsal margin sharply carinate, with two groups of spines, basal on at three quarters of metatibial length, apical one shortly before apex, basally with a few single spines; external face beside dorsal margin longitudinally roof-like carinate, densely coarsely punctate, with dense short setae; ventral margin finely serrate, with six robust equidistant setae; medial face coarsely and densely punctate, punctures each bearing a fine seta; apex moderately truncate interiorly near tarsal articulation. Tarsomeres densely punctate dorsally, with sparse, short setae ventrally; metatarsomeres with a strongly serrated ridge ventrally, dorsal punctures partly extended to longitudinal wrinkles; first metatarsomere distinctly longer than second, slightly longer than dorsal tibial spur. Protibia moderately long, bidentate, protarsal claws symmetrical.

#### Variation.

Length: 7.8–8.8 mm, length of elytra: 5.8–6.6 mm, width: 4.2–5.0 mm.

Female: Antennal club with three antennomeres, as long as remaining antennomeres combined; eyes nearly as large as in male (ratio of diameter/interocular width: 0.55).

#### Etymology.

The new species is named after the type locality in Mupu Mountain.

### Paraserica wangi

Taxon classificationAnimaliaColeopteraScarabaeidae

Ahrens, Fabrizi, & Liu sp. n.

http://zoobank.org/9FFF2588-4633-4288-9E4A-66D641121519

Figures 3I–L , 5

#### Type material examined.

Holotype: “Kuankuoshui Nature Reserve, Guizhou, 5.VI.2010, leg. Wang Zhiliang/ LW-355” (IZAS).

#### Diagnosis.

*Paraserica wangi* has longer phallobasal processes and the median interior lobe is directed distally.

#### Description.

Length: 8.3 mm, length of elytra: 5.8 mm, width: 4.3 mm. *Body* oblong, colour greyish-black, antenna yellowish brown, dorsal and ventral surface shiny and densely setose with partly double pilosity.

*Labroclypeus* subtrapezoidal and wide, widest at base; lateral margins straight and weakly convergent, with moderately rounded anterior angles; lateral border and ocular canthus producing a distinct blunt angle; margins weakly reflexed; anterior margin distinctly sinuate medially; surface flat and moderately shiny, densely punctate, very coarse punctures mixed with small ones; with long, dense, erect setae. Frontoclypeal suture distinctly incised and weakly curved medially, slightly elevated; smooth area in front of eye 1.5 times as wide as long; ocular canthus moderately short and narrow, finely and densely punctate, with a 1–2 setae. Frons with fine and dense punctures, with numerous short, adjacent setae and a few erect longer ones beside eyes and behind frontoclypeal suture. Eyes very small, ratio of diameter/interocular width: 0.46. Antenna with nine antennomeres; club with three antennomeres, 2.5 times as long as remaining antennomeres combined, strongly reflexed. Mentum elevated and flattened anteriorly. Labrum weakly produced and moderately sinuate medially.

*Pronotum* narrow, widest at base, lateral margins straight and subparallel in basal half, weakly convex and moderately convergent in anterior half; anterior angles weakly produced but nearly blunt; posterior angles right-angled; anterior margin weakly convex, with a distinct and broad marginal line; basal margin without marginal line; hypomeron distinctly margined at base; surface with dense and fine punctures, with dense, double pilosity: numerous short setae bent backwards mixed with sparse, long and erect setae; anterior and lateral borders densely setaceous. Scutellum narrow and long, with fine and dense punctures, with dense short setae.

*Elytra* oblong, widest at middle, striae distinctly impressed, finely and densely punctate; intervals weakly convex, with fine and sparse punctures, densely setose with short adjacent setae as pronotum, with numerous long erect setae on odd intervals; epipleural border robust, ending at strongly curved external apical angle; epipleura densely setaceous; apical border chitinous with a broad rim of short microtrichomes (visible at 100x magnification).

*Ventral surface* shiny, with moderately dense, fine punctures, with dense short adjacent setae. Metacoxa completely finely setose as rest of ventral surface, laterally with a few longer setae. Abdominal sternites with fine, dense punctation and short fine pilosity, each sternite with a distinct transversal row of coarse punctures bearing a long seta; penultimate sternite simple. Mesosternum between mesocoxae as wide as mesofemur, with irregularly scattered, strong setae. Ratio of length of metepisternum/metacoxa: 1/1.65. Pygidium shiny, beside apical margin dull, moderately convex, finely and densely punctate, without smooth midline, with dense, moderately long setae and numerous longer setae adjacent to apical margin.

*Legs* moderately slender, shiny; femora with two longitudinal rows of setae, finely and moderately densely punctate, shortly sparsely setose. Metafemur shiny, anterior margin acute, without a submarginal serrated line; posterior margin with a few strong setae medially, weakly widened in apical half and smooth ventrally; finely serrated dorsally. Metatibia slender and long, widest shortly before apex, ratio width/length: 1/3.6; dorsal margin sharply carinate, with two groups of spines, basal on at three quarters of metatibial length, apical one shortly before apex, basally with a few single spines; external face beside dorsal margin longitudinally roof-like carinate, densely coarsely punctate, with dense short setae; ventral margin finely serrate, with five robust equidistant setae; medial face coarsely and densely punctate, punctures each bearing a fine seta; apex moderately truncate interiorly near tarsal articulation. Tarsomeres densely punctate dorsally, with sparse, short setae ventrally; metatarsomeres with a strongly serrated ridge ventrally, dorsal punctures partly extended to longitudinal wrinkles; first metatarsomere distinctly longer than second, slightly longer than dorsal tibial spur. Protibia moderately long, bidentate, protarsal claws symmetrical. Female unknown.

#### Etymology.

The new species is named after the collector of this species, Wang Zhiliang.

**Figure 4.**
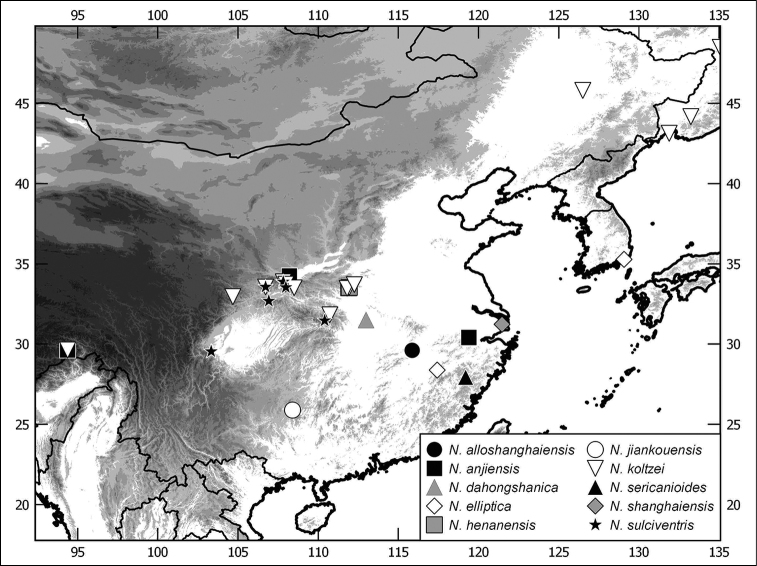
Distribution of Chinese *Nipponoserica* species (in case of *N. koltzei and N. elliptica* only material examined in this work is included).

**Figure 5.**
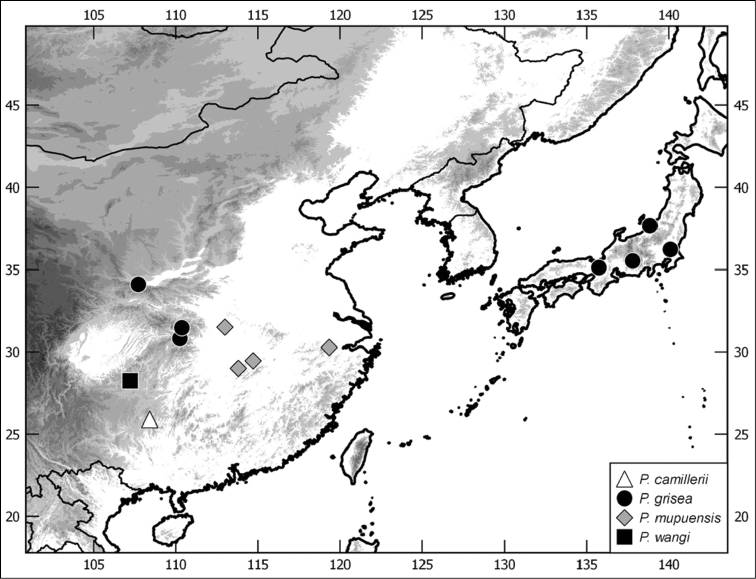
Distribution of Chinese *Paraserica* species (in case of *P. grisea* only material examined in this work is included).

## Supplementary Material

XML Treatment for Nipponoserica

XML Treatment for Nipponoserica elliptica

XML Treatment for Nipponoserica koltzei

XML Treatment for Nipponoserica sulciventris

XML Treatment for Nipponoserica anjiensis

XML Treatment for Nipponoserica alloshanghaiensis

XML Treatment for Nipponoserica henanensis

XML Treatment for Nipponoserica jiankouensis

XML Treatment for Nipponoserica sericanioides

XML Treatment for Paraserica

XML Treatment for Paraserica grisea

XML Treatment for Paraserica taiwana

XML Treatment for Paraserica camillerii

XML Treatment for Paraserica mupuensis

XML Treatment for Paraserica wangi
